# The Relevance of Gynecologic Oncologists to Provide High-Quality of Care to Women with Gynecological Cancer

**DOI:** 10.3389/fonc.2015.00308

**Published:** 2016-01-14

**Authors:** Lucas Minig, Pablo Padilla-Iserte, Cristina Zorrero

**Affiliations:** ^1^Gynecology Department, Valencian Institute of Oncology (IVO), Valencia, Spain

**Keywords:** gynecologic oncologists, ovarian cancer, vulvar cancer, endometrial cancer, cervical cancer, centralization of care, fellowship-training program

## Abstract

Gynecologic oncologists have an essential role to treat women with gynecological cancer. It has been demonstrated that specialized physicians who work in multidisciplinary teams to treat women with gynecological cancers are able to obtain the best clinical and oncological outcomes. However, the access to gynecologic oncologists for women with suspected gynecological cancer is scarce. Therefore, this review analyzes the importance of specialized care of women with ovarian, cervical, and endometrial cancer. In addition, the role of gynecologic oncologists who offer fertility-sparing treatment as well as their role in assisting general gynecologists and obstetricians is also reviewed.

## Introduction

It is estimated that over a million new cases and half million deaths are due to gynecological cancers that occur annually worldwide (1). Even though general gynecologists commonly treat these diseases across the world, the sub-specialization in gynecologic oncology has been progressively increasing in developed countries since 1972 (2, 3).

According to the definition of the American Board of Obstetrics and Gynecologist, gynecologic oncologist is “a specialists in obstetrics and gynecology who is prepared to provide consultation on comprehensive management of patients with gynecologic cancer and who works in an institutional setting wherein all the effective forms of cancer therapy are available” (4).

Gynecologic oncologists have an essential role when treating women with gynecological cancer. They are in a unique position to enrich the global health community with opportunities for education, training, and policymaking as it pertains to women’s cancers. In addition, they are in a privileged position to make decisions regarding the integration and sequencing of all modalities of treatment.

Specialized physicians who work in multidisciplinary teams to treat women with gynecological cancers obtain the best clinical and oncological outcomes (5–7). We think that by this approach, gynecologic oncologists not only play an important role in performing an optimal surgery but they can also provide a better overall quality of care by having a holistic conception of women. However, in countries with a high number of gynecologic oncologists, only a minority of women with gynecological cancer receives care by specialized physicians at referral institutions (8). For example, it has been demonstrated that approximately one-third of women with ovarian cancer are treated by gynecologic oncologists in the U.S. Therefore, this article will review the role of gynecologic oncologists who treat women with different gynecological malignancies.

## Ovarian Cancer

Ovarian cancer probably represents the best example of how a well-prepared specialist can positively modify the clinical and oncologic outcomes of women. Ovarian cancer is the most aggressive gynecological cancer with a 5-year overall survival of 40% (9). There are well-documented independent prognostic factors at advanced-stage disease, including tumor histology and grade of differentiation, patient’s age, stage of disease, performance status, and surgical debulking (10). However, the latter is the only modifiable factor, which means that it is amenable for direct influence, and therefore, seems to be of the utmost importance when considering efforts aiming toward improving outcomes of this disease.

The relevance of an adequate surgery was highlighted in multiple studies, which associated a significant improvement in oncological outcomes after a complete tumor resection at the time of primary surgical cytoreduction in comparison with cases in which there was some amount of residual disease at the end of the surgical procedure (9–11). Thus, according to the last Gynecological Cancer InterGroup (GCIG) consensus conference, “the mainstay of treatment of advanced ovarian cancer is primary surgery aiming at complete tumor resection followed by platinum and paclitaxel chemotherapy” (12).

However, the final decision as to whether or not to perform a tumor debulking depends on the surgeon’s training and confidence (13). Many studies suggest that patients operated on by gynecologic oncologists with previous training in cytoreductive techniques are more likely to undergo an adequate surgical staging in the early stage of the disease, and a better rate of complete cytoreduction in advanced stages in comparison with those patients treated by general gynecologists or general surgeons (5–7). More specifically, when gynecologic oncologists perform the surgery, there are twice as many probabilities of obtaining a complete cytoreduction (5). As a consequence, according to the results of meta-analyses, patients operated on by gynecologic oncologists have significantly better oncological outcomes, which resulted in an increased overall survival of 10 months, in comparison with those patients treated by general gynecologist or general surgeons (5–7).

A recent document launched by the European Society of Gynecological Oncology (ESGO) regarding the quality indicators in ovarian cancer surgery states that “Surgery is performed by a certified gynecologic oncologist or, in countries where certification is not organized, by a trained surgeon dedicated to the management of gynecologic cancer (accounting for over 50% of his practice) or having completed an ESGO accredited fellowship. Skills to successfully complete abdominal and pelvic surgery procedures necessary to achieve complete cytoreduction must be available” (14).

**Figure 1:**
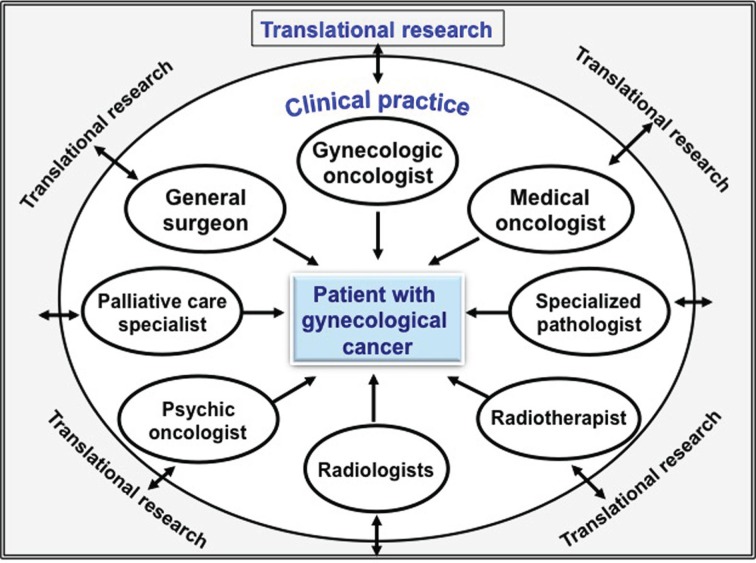
**Multidisciplinary team to treat women with gynecological cancer**.

However, ovarian cancer surgery is not an easy task, and it requires an adequate institutional support, as well as establishing evidence-based clinical guidelines. Even though gynecologic oncologists should lead these surgeries, it is recommended to work in a multidisciplinary surgical team involving other specialists, such as general surgeons, anesthesiologists, and infectologist. This strategy is aimed to offer the best quality of care for the patient (Figure 1).

## Endometrial Cancer

The majority of endometrial cancers are low-risk disease with excellent oncological outcomes (15). Thus, the potential positive impact of subspecialty care in endometrial cancer might be more difficult to demonstrate (16). However, gynecologic oncologists can have an important role in the implementation of minimally invasive surgery with their well-known advantages over open surgery (17). In this regard, a recent U.S. epidemiological study demonstrated that 86% of robotic surgeries for endometrial cancer were performed in 19% of the analyzed hospitals. Each additional 25 patients (above the mean surgical volume) were associated with over a 2.5-fold increase in odds of robotic surgery (OR = 2.65, 95% CI: 1.82–3.86; *p* < 0.0001) (17).

In addition, another epidemiology study performed in the U.S. evaluated 18,338 women with endometrial cancer, 4,489 of whom at stages II–IV (24.3%) (18). Patients who underwent surgery by a gynecologic oncologist were more likely to receive a more extensive lymph node resection (16 lymph nodes; 22 vs. 17%; *p* < 0.001), have more aggressive histologic cell types, such as serous and clear-cell (11.6 vs. 6.1%; *p* < 0.001), presented with advanced-stage disease (stages III and IV; 21.9 vs. 14.6%; *p* < 0.001), were more likely to received chemotherapy (22.6 vs. 12.4%; *p* < 0.001), as well as to received radiotherapy (38.9 vs. 30.7%; *p* < *0*.001). In addition, surgery performed by gynecologic oncologist was an independent prognostic factor and was associated with a 30% increase in overall survival in comparison with other type of surgeons (HR, 0.71; 95% CI, 0.62–0.82; *p* < 0.001) (18). Other studies did not find significant differences in the survival rate, probably because they only included early-stage disease (19), or due to the fact that they only analyzed a small number of patients (16, 19).

## Cervical Cancer

Worldwide, cervical cancer accounts for over 500,000 cases and 275,000 deaths each year (20). However, there is a great disparity among high- and low-income countries due to successful implementation of cervical cancer screening programs in developed countries (21). In addition, the reduction in the incidence of cervical cancer should continue with the increased use of human papilloma virus (HPV) vaccination (22). Therefore, the majority of women with cervical cancer in developed countries are diagnosed at early stages with a 5-year overall survival rate of over 90% (23).

No studies have specifically addressed the impact on survival of women with early-stage cervical cancer treated by gynecologic oncologists. One U.S. epidemiological study, however, studied 27,660 women with cervical cancer FIGO stage IIB–IVB who were treated at hospitals with different case volumes. The study showed that the median rate of survival of patients treated at the lowest and highest volume centers were 42.3 months (95% CI 39.8–44.8) and 53.8 months (50.1–57.5), respectively (*p* < 0.001). On multivariable analysis, higher facility volume independently predicted improved survival (*p* = 0.022), increased likelihood of receiving brachytherapy (*p* < 0.0005) and chemotherapy (*p* = 0.013), as well as shorter time to radiotherapy completion (*p* < 0.0005) (24).

## Vulvar Cancer

Squamous cell cancer of the vulva is a rare disease with an annual incidence of 2–3/100,000 women (25). There is evidence that demonstrates step-by-step nodal metastases in human cancer (26). Therefore, the first regional lymph node, called sentinel node, receiving lymphatic fluid from the tumor is initially removed. All regional lymph nodes are only dissected in case of disease in the sentinel node. Thus, this technique significantly reduces the incidence of postoperative complications, such as wound breakdown or cellulitis, and long-term morbidity including lymphedema (27). However, failure in the sentinel node detection is mainly seen when specialists with low case-volume (<10 cases/year) perform the procedure (27). Failure of this procedure can mean leaving the sentinel node in place, probably with tumor cells and with fatal consequences for patients. Therefore, some authors recommend that sentinel node detection in patients with vulvar cancer should be offered to well-selected patients by well-trained and informed gynecologic oncologists who work in centers with at least 10 cases/year (27, 28). In addition, it is also recommended that this technique be performed by multidisciplinary team involving gynecologic oncologists, specialists in nuclear medicine, and specialized pathologists (27).

## Role of Gynecologic Oncologists in Special Circumstances

### Fertility Preserving Treatment in Women with Gynecological Cancers

It is estimated that over 21% of women with gynecological cancer are diagnosed in their reproductive age (29). In addition, it has been demonstrated a continuum increase of women age at first pregnancy (30, 31). Both factors explain why fertility preservation in women with gynecological cancer is currently a very important issue. The recommended treatment for the great majority of gynecological cancer includes radical removal of the uterus and ovaries, annulling any possibility for future pregnancies. However, fertility-sparing treatment in young patients with women’s cancer is possible in very select women without compromising long-term survival (32). A recent survey of the ESGO revealed that only a minority of young women candidates to fertility-sparing treatment is aware of the opportunity to preserve their fertility (33). The main reasons include the surgeon’s being unaware, skeptic, or untrained to perform fertility-sparing surgical procedures (33). Despite the fact that fertility-sparing surgery is technically not difficult (except for radical trachelectomy for cervical cancer), a more complicated task can be to select the appropriate candidate for such specific treatment. Therefore, according to an ESGO statement, these patients should be managed in a multidisciplinary team coordinated by gynecologic oncologists in conjunction with medical reproductive endocrinologists, perinatologists, pathologist, psychologists, and assisted reproductive specialists (33).

### Surgical Assistance to General Gynecologists and Obstetricians

Even though gynecologic oncologists are intensively trained in all aspects of women’s cancer care, their main area of expertise is focused on performing complex surgical procedures. Therefore, their role in clinical practice often extends beyond women’s cancer. For instance, they can be of assistance to general gynecologists/obstetricians at certain moments during difficult surgical procedures, such as being a surgical resource to obstetricians during challenging peripartum hemorrhage, (34–36) as well as in cases of complex pelvic anatomy or pathological placentation (37). A recent study performed at Massachusetts General Hospital revealed that gynecologic oncologists can assist general gynecologists at the time of intraoperative consultation in 98 out of 794 benign gynecological surgical procedures (12.3%). The main reasons for unplanned consultation included adhesive disease, bowel injury, ureter visualization, cancer, and bleeding control (34).

## Centralization of Care – Multidisciplinary Management

Gynecological cancer is a challenging, complex, and multidisciplinary disease. It is not only important how well trained the physicians are but also how many physicians of different specialties are involved (38). The concept of the holistic conception of patient care under a multidisciplinary team approach is crucial from the diagnosis to the demise of the disease, and this model should not be restricted to the operating room setting.

**Figure 2:**
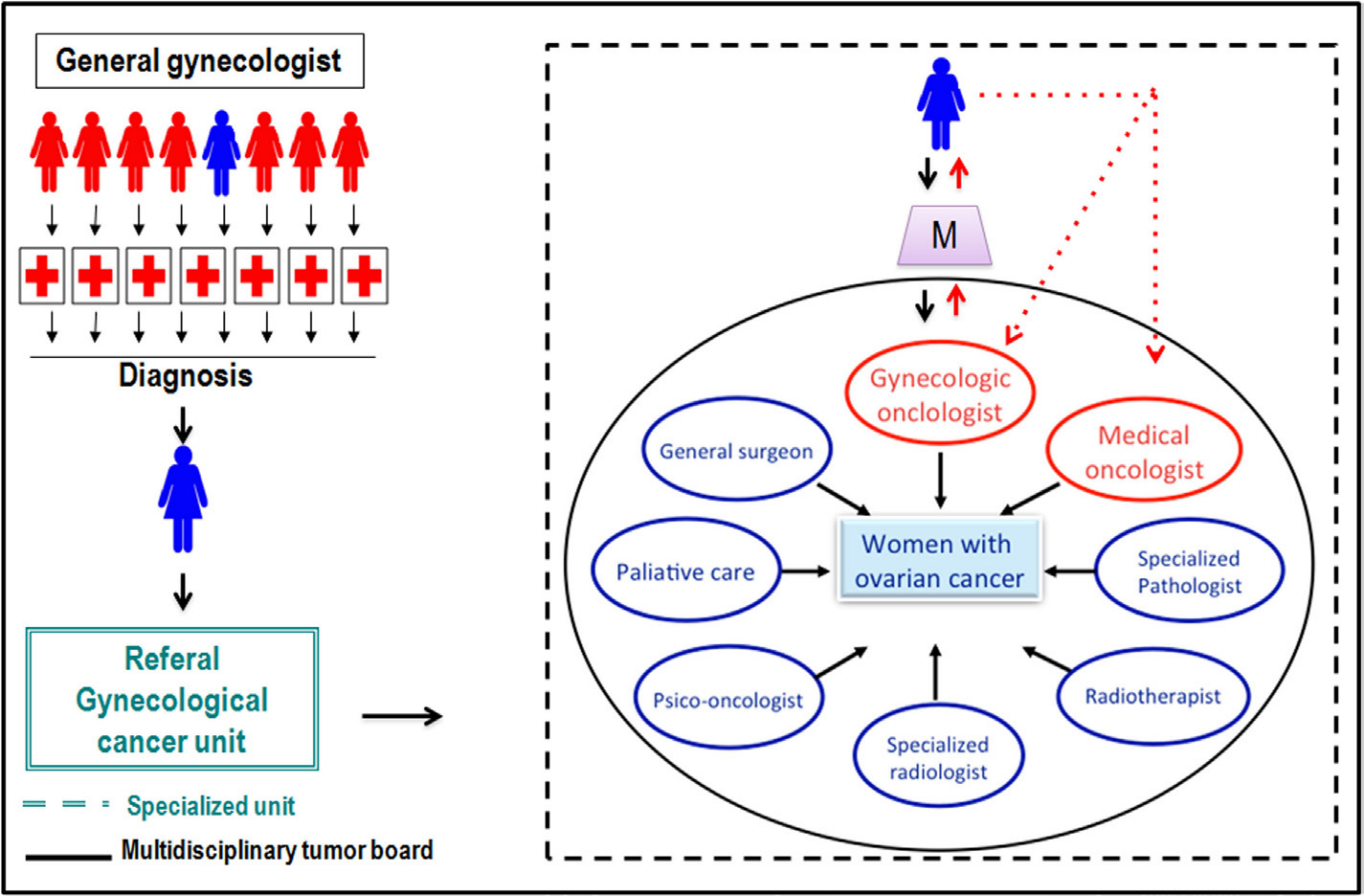
**Multidisciplinary care in a gynecological cancer unit**.

A correct collaboration with dedicated pathologists, medical oncologists, radiotherapists, biologists, palliative care specialists might help avoid unnecessary mismanagements, and therefore, reinforce the holistic conception of patients with gynecological cancer with an improvement on their perceived quality of care. Moreover, the recent molecular biology, genetic, and immunology discoveries are opening new optimistic frontiers for the future treatment and cure of this disease. Many authors agree that close exchanges between the clinical practice and basic research are crucial for consolidating these progresses (39, 40) (Figure 2). A recent meta-analysis demonstrated that women with gynecological malignancies who receive care from a multidisciplinary team by specialized physicians live for a significantly longer period of time (7).

Centralization of care in women with gynecological cancer is another crucial issue. In some regions, gynecologic oncology cases have been centralized (41) in centers with higher patient volumes and interdisciplinary collaboration (42). These centers receive referrals from less-specialized hospitals within a network, region, or defined catchment area. Under this model of care, women are referred to specialized units, which are a team built by multiple specialized physicians focused on the comprehensive care of women affected by gynecological cancer. Every case is discussed inside in a multidisciplinary tumor board where the best strategy of treatment is based on multiple points of views, taking into consideration all aspects regarding each individual patient expectancy beyond the disease itself (Figure 2).

Results of different studies consistently show that patients with ovarian cancer treated at high-volume hospitals receive better quality of care, which is accomplished by better surgical staging and better optimal cytoreduction rate (5–7), as well as better chemotherapy administration rate and schemes (43, 44).

One study, performed in England, showed that the survival of patients with gynecologic cancer improved significantly after centralization in comparison with the pre-centralization period (hazard ratio: 0.71; 95% confidence interval: 0.64–0.79) (45). Similar findings were also reported for cervical, endometrial, and ovarian cancer after the implementation of the U.K. National Health Service cancer plan in 2000 (46).

Despite the consensus recommendations (12) and the advantages previously explained, population-based studies indicate that access to specialist care in gynecologic oncology for women with suspected gynecological cancer is uncommon (47–49). Reports from countries such as U.S. (8, 50) and U.K. (49, 51) have showed that over 60–80% of patients with advanced stage ovarian cancer are treated in low-volume hospitals by low-volume surgeons (8, 52, 53).

Common barriers to the quality of cancer care have been identified by multiple investigators and include the extremes of age, minority race, low socioeconomic status, rural residence, patient’s and physician’s unawareness of gynecologic oncologist resources, ineffective recognition of the disease, and third-party payers (8, 47).

## Fellowship-Training Program

Gynecologic oncologists have also an important role in teaching and educating fellow colleagues. Physicians who want to be gynecologic oncologists need to undergo a long and specific period of training and education process. After finishing medicine school, physicians must complete a 4- to 5-year residency-training program in obstetrics and gynecology. Then, they need to be accepted in accredited referral institutions to complete their specific fellowship-training program for 2–4 more years. The training, skills, and knowledge base required of a gynecologic oncologist are rapidly expanding. In addition to the original areas of radical pelvic surgery, chemotherapy, radiation therapy, and pathology, new areas of training include radical upper abdominal surgery, minimally invasive and robotic surgery, translational medicine and research, and palliative medicine (54).

In 1972, the first gynecologic oncologic fellowship-training programs were introduced in the U.S. with two accredited fellowships. Since then, 46 fellowship programs exist with 126 approved positions (55). Currently, there is a uniform system of training developed by the American Board of Obstetrics and Gynecologist who provide the training resource documents for the development of a curriculum in gynecologic oncology (4). Australia, Canada, U.K., and recently, the European Union are other examples of renowned gynecologic oncologic fellowship-training programs around the world (2). However, the number of gynecologic oncologists per patient is still scarce worldwide, and it is expected that the number of fellowship positions will continue to increase through the following years (56).

## Conclusion

When women with gynecological cancers are treated by gynecologic oncologists in referral cancer centers, they are able to live longer and with a better quality of life. Therefore, patients should be ideally referred to high-volume physicians/hospitals to increase their life expectancy as well as its quality. Expanding fellowship-training programs worldwide as well as highlighting the existence and relevance of gynecologic oncologists in the general population and medical community is crucial to increase the patient’s accessibility to a specialist’s care.

## Author Contributions

All of the three authors of the present paper declare that there are no conflicts of interest and have actively participated in the study providing input including (1) substantial contributions to conception and design, or acquisition of data, or analysis and interpretation of data; (2) drafting of the article, or provision of critical revision for important intellectual content; and (3) final approval of the version to be published.

## Conflict of Interest Statement

The authors declare that the research was conducted in the absence of any commercial or financial relationships that could be construed as a potential conflict of interest.
